# Effect of Comorbidity on Lung Cancer Diagnosis Timing and Mortality: A Nationwide Population-Based Cohort Study in Taiwan

**DOI:** 10.1155/2018/1252897

**Published:** 2018-11-04

**Authors:** Shinechimeg Dima, Kun-Huang Chen, Kung-Jeng Wang, Kung-Min Wang, Nai-Chia Teng

**Affiliations:** ^1^School of Dentistry, College of Oral Medicine, Taipei Medical University, 250 Wu-Hsing Street, Taipei, Taiwan; ^2^National Taiwan University of Science and Technology, Taipei, Taiwan; ^3^Big Data Research Center, Asia University, Taichung, Taiwan; ^4^Department of Surgery, Shin-Kong Wu Ho-Su Memorial Hospital, Shilin District, Taipei 111, Taiwan; ^5^Department of Dentistry, Taipei Medical University Hospital, 250 Wu-Hsing Street, Taipei, Taiwan

## Abstract

The effect of comorbidity on lung cancer patients' survival has been widely reported. The aim of this study was to investigate the effects of comorbidity on the establishment of the diagnosis of lung cancer and survival in lung cancer patients in Taiwan by using a nationwide population-based study design. This study collected various comorbidity patients and analyzed data regarding the lung cancer diagnosis and survival during a 16-year follow-up period (1995–2010). In total, 101,776 lung cancer patients were included, comprising 44,770 with and 57,006 without comorbidity. The Kaplan–Meier analyses were used to compare overall survival between lung cancer patients with and without comorbidity. In our cohort, chronic bronchitis patients who developed lung cancer had the lowest overall survival in one (45%), five (28.6%), and ten years (26.2%) since lung cancer diagnosis. Among lung cancer patients with nonpulmonary comorbidities, patients with hypertension had the lowest overall survival in one (47.9%), five (30.5%), and ten (28.2%) years since lung cancer diagnosis. In 2010, patients with and without comorbidity had 14.86 and 9.31 clinical visits, respectively. Lung cancer patients with preexisting comorbidity had higher frequency of physician visits. The presence of comorbid conditions was associated with early diagnosis of lung cancer.

## 1. Introduction

Lung cancer is the most commonly diagnosed cancer worldwide and places a considerable burden on public health [1, 2]. Lung cancer incidence and mortality exceed that of any other cancer globally, with 1.6 million cases (accounting for 12.7% of total cancer incidence) and 1.4 million deaths occurring in 2008 worldwide [3]. According to the 2011 Taiwan Cancer Registry annual report, lung cancer was the third most common diagnosed cancer accounting for 13% of all cancers among men and 10% among women in Taiwan [4]. Despite advances in treatment, lung cancer survival has remained relatively poor because most patients are diagnosed as having lung cancer at an advanced stage [5]. Specific comorbidities have been recognized as being highly prevalent in lung cancer patients, including chronic obstructive pulmonary disease (COPD), hypertension, cardiovascular disease, diabetes mellitus (DM), and other malignancies [6, 7], with a prevalence of 26.4%–81.2% [8].

Asthma, COPD, and tuberculosis (TB) are the most common pulmonary comorbidities. However, studies regarding the effects of these comorbidities on lung cancer survival have included small samples sizes and yielded conflicting results. In a cohort of 1155 patients, Tammemagi et al. [9] reported that 18 of 56 comorbidities, including asthma, COPD, TB, and pulmonary fibrosis, were associated with a reduced lung cancer survival. In a cohort of 5406 patients, preexisting pulmonary diseases increased mortality risk in male patients with squamous cell carcinoma, whereas preexisting asthma reduced mortality in female patients with early-stage squamous cell carcinoma [10]. Kuo et al. [11] reported that compared with those without comorbidity, non-small-cell lung cancer (NSCLC) patients with concomitant active TB had a more favorable survival outcome, particularly those with squamous cell carcinoma. Furthermore, patients with bronchiectasis, a chronic inflammatory airway disease, have an increased risk of lung cancer compared with the general population [12]; however, studies regarding the effects of bronchiectasis and other pulmonary diseases on lung cancer survival are scarce.

Despite increasing recognition of the impact of comorbidities on the prognosis of cancer patients, challenges remain. Population-based cohort studies are preferable for a detailed evaluation of the association between comorbidity and lung cancer. This nationwide population-based study explored the effects of comorbidity on lung cancer diagnosis timing and survival in lung cancer patients in Taiwan.

## 2. Methods

### 2.1. Database

In this study, we obtained patient data from the National Health Insurance Research Database (NHIRD), derived from the National Health Insurance (NHI) program. The NHIRD contains all medical claims for inpatient and ambulatory care services, registry files of contracted medical facilities, board-certified specialists, other medical service providers, and prescriptions covered by the NHI program for 25.68 million enrollees in Taiwan. The NHIRD data is deidentified secondary data, released to the public for research purposes by the Taiwan National Health Research Institutes.

This study was approved by the institutional review board of Shin-Kong Wu Ho-Su Memorial Hospital, Taiwan (20140703R). The study was conducted in accordance with the regulations of National Health Research Institute of Taiwan. The NHIRD is protected by computer-processed personal data protection act and does not include any personal data of enrollees. Therefore, no informed consent was obtained during this study.

### 2.2. Study Patients and Design

We employed a retrospective cohort to investigate the effect of comorbidity on the establishment of the diagnosis of lung cancer and survival. The lung cancer patients included in this study were identified from the NHIRD by using the International Classification of Diseases, 9th Revision, Clinical Modification (ICD-9-CM) code 162. The study population comprised lung cancer patients with and without comorbidity, selected from complete NHI claims database in the period 1995–2010.

**Figure 1:**
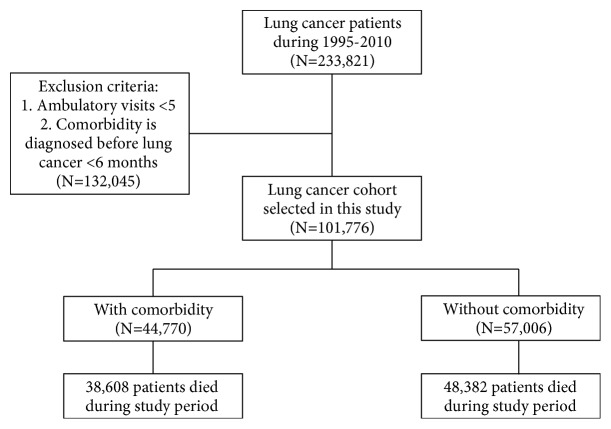
Flowchart of study population selection.

In total, 101,776 lung cancer patients were included, the comorbidity group comprised 44,770 patients who received a principal diagnosis of comorbidity (under ICD-9-CM) during ambulatory medical care visits between January 1, 1995, and December 31, 2010, at least 6 months before the lung cancer diagnosis. The index visit was defined as the first ambulatory visit, during which a principal diagnosis of comorbidity was made. To maximize case ascertainment, only patients with at least five ambulatory visits (including the index visit) and who received their principal diagnosis of comorbidity in this period were included in the comorbidity group. The noncomorbidity group comprised the remaining patients from the NHI claims database diagnosed as having lung cancer during 1995–2010; in total, 57,006 patients were included in the noncomorbidity group. Details were described in the Figure 1.

All ambulatory medical care records and inpatient records for each patient in both groups were tracked since their index visit for lung cancer. The date of the first principal diagnosis of lung cancer within the follow-up period was defined as the primary endpoint in cancer diagnosis timing analysis. The mortality data of the patients who died during the follow-up period was subsequently considered to determine the survival of lung cancer patients. Patients newly diagnosed as having lung cancer during 1995–2010 were followed up until death, loss to follow-up, or the study endpoint in 2010.

### 2.3. Comorbidities

The 15 most prevalent chronic conditions in Taiwan are hepatitis, cancer, diabetes, hyperlipidemia, gout and other crystal arthropathies, depression, eye disorders, nervous system disorders, hypertension, heart disease, cerebrovascular disease, respiratory disease, digestive disease, genitourinary disease, and joint disorder. We selected common chronic diseases which probably have association with lung cancer for evaluation. The following individual comorbidities were identified using ICD-9-CM codes for exploring their effects on lung cancer survival: cerebrovascular accident, coronary artery disease, diabetes mellitus (DM), disorders of adrenal gland, disorders of thyroid gland, duodenal ulcer, gastric ulcer, gastrojejunal ulcer, hyperlipidemia, hypertension, hypoparathyroidism, peptic ulcer, asthma, bronchiectasis, chronic bronchitis, emphysema, empyema, and pulmonary tuberculosis. The detailed information of ICD-9-CM codes used to identify comorbidities were described in Table 1.

### 2.4. Variables

The data on patients' gender, lung cancer diagnosis period, age at lung cancer diagnosis, geographical region, and residence urbanization level were retrieved from the NHIRD. On the basis of their age at lung cancer diagnosis, the patients were classified into five age groups: ≤39, 40–49, 50–59, 60–69, and ≥70 years. According to the cancer diagnosis period, the patients were classified into three groups: 1995–2000, 2000–2005, and 2006–2010. We then geographically divided Taiwan into northern, central, southern, eastern, and islands regions. On the basis of the population density, manufacturing industries, number of physicians per 1000 people, and availability of health care facilities, patients in each region were classified into three urbanization levels—from most (level 1) to least (level 5) urbanized. In addition, the 22 cities and counties of Taiwan were grouped into four regions: northern (Taipei, New Taipei, Hsinchu, Keelung, and Taoyuan cities; Yilan and Miaoli counties), central (Taichung city; Changhua, Nantou, and Yunlin counties), southern (Chiayi, Tainan, and Kaohsiung cities; Chiayi, Pingtung, and Penghu counties), and eastern (Hualien, Taitung, Kinmen, and Lienchiang counties) Taiwan and islands.

### 2.5. Statistical Analysis

Frequency distributions on demographic characteristics of death subjects among comorbidity and noncomorbidity groups were compared by the chi-square test. A two-tailed *p* value of <0.05 was considered statistically significant. The Kaplan–Meier method was used to calculate cumulative curves of lung cancer in patients with different comorbidities. The overall survival of lung cancer patients with and without comorbidity was calculated using the Kaplan–Meier method, and the difference in overall survival was determined using the log-rank test. All statistical analyses were performed using SAS version 9.4 (IBM, USA). The survival curves and cumulative proportion curves were plotted using SPSS version 20.0 (IBM, USA).

## 3. Results

**Figure 2:**
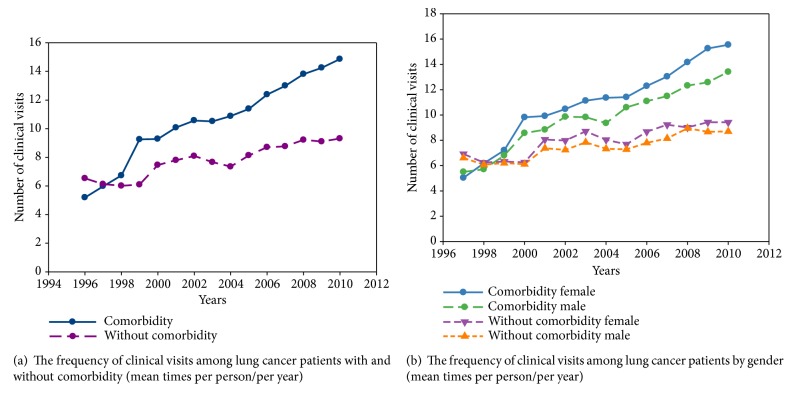


Of the 101,776 lung cancer patients enrolled in present study (mean age, 64.42 ± 13.22 years), 44% had comorbidities. Over the follow-up period, patients with comorbidity had a higher frequency of clinical visits than did those without comorbidity (Figures 2(a) and 2(b)). The mean number of clinical visits of patients with comorbidity increased from 5.19 per person in 1996 to 14.86 in 2010. By contrast, patients without comorbidity had 6.53 mean clinical visits per person in 1996, gradually increasing to 9.31 in 2010. The increase in frequency of clinical visits was observed in patients of both genders.

Table 2 presents the distribution of demographic characteristics by comorbidity presence in lung cancer death subjects. Results showed each characteristic to be strongly associated with comorbidity presence. Males accounted for higher mortality in both groups than females. For the period of diagnosis, death subjects without comorbidity were predominantly diagnosed in higher proportion before 2005 than subjects with comorbidity. With regard to diagnostic age, subjects without comorbidity were higher than subjects with comorbidity in age groups younger than 69. Except in most urbanized areas (level 1), death subjects with comorbidity were likely to reside in less urbanized areas. With regard to geographic region, northern Taiwan accounted for significantly higher proportion of residence in death subjects of both groups, followed by southern and central regions.

**Figure 3:**
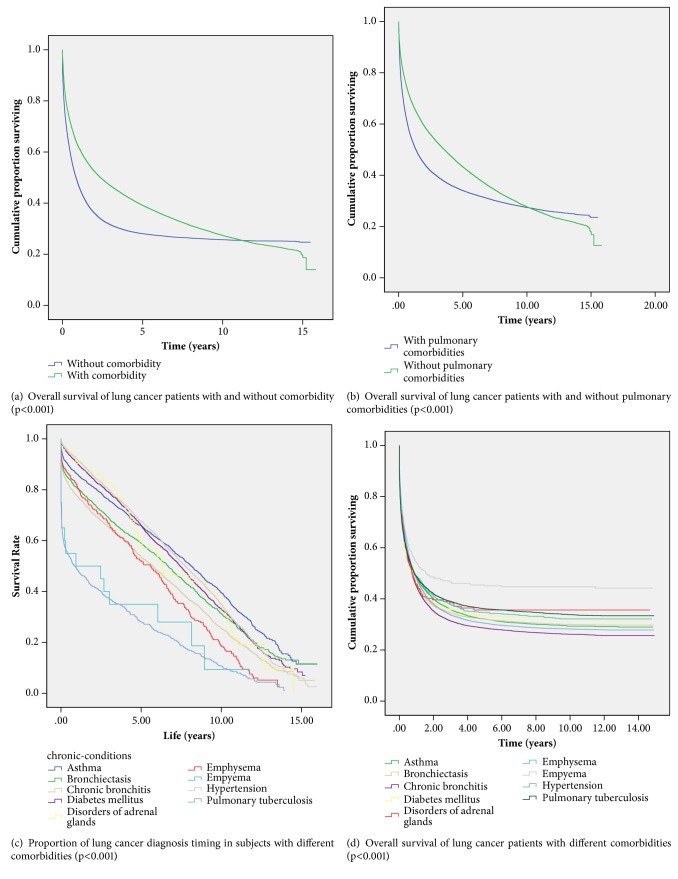
Kaplan–Meier survival curve of lung cancer patients with and without comorbidity over the 16-year follow-up period.

Figure 3 illustrates the Kaplan–Meier survival curve of lung cancer patients with and without comorbidity over the 16-year follow-up period. Patients without comorbidity had significantly lower 16-year survival than did those with comorbidity (*p *< 0.001) (Figure 3(a)).  Figure 3(b) presents the subgroup survival of comorbidity group by nature of comorbidity. The survival of lung cancer patients without pulmonary comorbidities was higher than that of those with pulmonary comorbidities (89%;* p *< 0.001).

Figure 3(c) presents the proportion and timing of lung cancer diagnosis with comorbidity.  Figure 3(d) depicts the survival analysis of the lung cancer patients with different comorbidities (*p*<0.001). In our cohort, chronic bronchitis patients who developed lung cancer had the lowest overall survival in one (45%), five (28.6%), and ten years (26.2%) since lung cancer diagnosis. Among pulmonary comorbidities, lung cancer patients with asthma had the second lowest overall survival with 48.5%, 32.1%, and 29.3% overall survival in one, five, and ten years since lung cancer diagnosis, respectively. The highest overall survival was observed in empyema patients with 54.1%, 45.1%, and 44.7% overall survival in one, five, and ten years since lung cancer diagnosis, respectively. Lung cancer patients with TB had higher overall survival (48.8%, 36.1%, and 33.9% in 1, 5, and 10 years since cancer diagnosis) compared to patients with other pulmonary comorbidities. Among lung cancer patients with nonpulmonary comorbidities, patients with hypertension had the lowest overall survival in one (47.9%), five (30.5%), and ten (28.2%) years since lung cancer diagnosis.

## 4. Discussion

Our findings of 101,776 patients showed that lung cancer patients with comorbidity had significantly superior overall survival compared with those without comorbidity. The potential factors contributing to this finding are discussed as follows.

Lung cancer is the most prevalent cause of cancer death. A reason for low lung cancer survival rates is the lack of observable symptoms in the early stages. Lung cancer diagnosed on the basis of advanced-stage symptoms has limited treatment options. In Taiwan, data from 2004 to 2008 demonstrated that more than half of the lung cancer cases were diagnosed at advanced stages (e.g., stage IV) [13]. The odds of being diagnosed with advanced-stage lung cancer is lowered in the presence of comorbidity [14–18]. Comorbidity is associated with lung cancer diagnosis at an earlier stage, whereas the absence of comorbidity is associated with lung cancer diagnosis at a later stage [19]. In their population-based study in Taiwan [20], Wang et al. reported that early-stage lung cancer patients had more favorable survival rates; the 5-year survival rates were 60.7%, 36.3%, 13.3%, and 4.9% for stages I, II, III, and IV lung cancer, respectively. On the basis of data from the Taiwan Cancer Registry, Chang et al. [21] reported increased survival of patients with lung adenocarcinoma in Taiwan, which is also attributable to EGFR inhibitors having been approved for treating adenocarcinoma since 2003 (Gefitinib) and 2007 (Erlotinib). The chance of earlier cancer diagnosis is increased through seeking care for the symptoms of comorbidities or during regular monitoring of comorbidities, potentially assisting in the timely administration of adequate curative measures. With respect to health care utilization, our results showed increase in health-seeking behavior in lung cancer patients with comorbidity compared with those without comorbidity. Our findings are reasonable from a clinical perspective because patients with comorbidity are more likely to require frequent medical care and clinical visits than are healthier patients, resulting in closer monitoring and detection of cancer at early stages. This phenomenon previously explained as screening bias [7] or the surveillance effect was also seen in other cancers. Several studies have revealed the diagnosis of breast cancer [22, 23] and colon cancer [24] at early stages is associated with higher comorbidity scores. This pattern has most commonly been reported for screen-detected cancers (breast and colorectal), supporting the contention that a higher number of clinical visits may be related to a higher number of screenings, particularly where screening coverage rates are associated with health service funding, potentially encouraging the screening of patients with high comorbidity scores.

For detailed investigation on the impact of comorbidity on lung cancer, we performed proportion and timing of diagnosis and survival analysis of lung cancer in patients with nine comorbidities. The results indicated that lung cancer patients with pulmonary diseases had lower overall survival than did those with nonpulmonary diseases. These results corroborated the analysis that patients with respiratory tracts compromised by cancer cannot tolerate additional pulmonary comorbidities as well as those with tumor-free lungs. However, further survival analysis revealed that patients with asthma and chronic bronchitis had lower overall survival than did those with other pulmonary comorbidities. Although previous studies have reported the effects of asthma and COPD on decreased survival in lung cancer, gender differences in the survival of patients with different lung cancer types and preexisting asthma or COPD were reported in Taiwan. A study that involved enrolling 5406 squamous cell carcinoma patients from Taiwan demonstrated a hazard ratio for mortality of 1.08 and 1.04 for participants with asthma and COPD, respectively. Furthermore, female stage I and II squamous cell carcinoma patients with preexisting asthma had a hazard ratio of 0.19, suggesting decreased mortality for these patients [10]. In another study including 13,399 patients with lung adenocarcinoma, the hazard ratios for male adenocarcinoma patients with asthma and COPD were 1.20 and 1.32, whereas for female patients they were 1.05 and 0.97, respectively. In particular, male patients with lung adenocarcinoma and preexisting pulmonary diseases exhibited an increased mortality risk, whereas female patients demonstrated no change in mortality risk [25]. Therefore, the burden of preexisting pulmonary comorbidities and their effects on survival of specific lung cancer types should be investigated further.

In addition, our analyses of lung cancer establishment of the diagnosis in comorbidity patients revealed that time period for lung cancer diagnosis in tuberculosis patients was shorter than those with asthma and COPD. Previous findings reported higher HRs for tuberculosis than for asthma and COPD [26]. The survival in those patients with coexisting TB and lung cancer remains inconclusive [11, 27]. We found lung cancer patients with preexisting TB had higher overall survival than those with other pulmonary comorbidities. In this study, the overall survival of lung cancer patients with preexisting adrenal glands disorder was 35.6% after ten years since cancer diagnosis. Currently, the data required for exploring the association between adrenal gland disorders and lung cancer are limited. The relevant literature indicates that approximately 30% of all small-cell lung cancer (SCLC) cases are associated with the hypersecretion of adrenocorticotrophic hormone (ACTH) [28]. Patients with SCLC associated with ectopic ACTH production exhibit low responses to chemotherapy and high rates of complication to therapy [29], which may shorten survival. The management of these patients is extremely complex. Future studies should focus on establishing the mortality risk of lung cancer in these patients. In this study, overall survival of lung cancer patients with bronchiectasis was 32% and 30% after five and ten years since cancer diagnosis, respectively. Only few studies have examined the association between lung cancer and noncystic fibrosis bronchiectasis, a representative chronic airway inflammatory disease. A cohort study in Taiwan reported that patients with bronchiectasis had a 2.36-fold higher risk of lung cancer than did the general population [12]. Kim et al. observed that the concomitant presence of bronchiectasis in advanced COPD patients was associated with lower risk of lung cancer [30]. This is a crucial area for future research, given the potential for learning more on reported protective effects of concomitant bronchiectasis on lung cancer diagnosis timing and survival.

In Taiwan, after the implementation of the NHI program in 1995, the number of diagnosed cancer cases increased, with lung cancer being one of 10 leading cancers [31]. Present study found geographic and urbanization variations in lung cancer mortality across Taiwan. In comparison to China, where rural areas with limited health care resources have higher cancer incidence and mortality [32], we found higher mortality among patients residing in urbanized areas of northern, central, and southern Taiwan compared with those in rural areas. Notably, the most high polluting industries, such as the petroleum, petrochemical, iron, steel, pig farming, and energy industries, are located in the areas of central [33] and southern [34] Taiwan. Moreover, a higher incidence of lung cancer has been reported in the endemic arseniasis areas of southwestern [35] and northeastern Taiwan [36, 37].

Following the promulgation of the Cancer Control Act in 2003, a 5-year national cancer control program was implemented in 2005 in Taiwan. Subsequently, the incidence of certain cancers, including cervical, stomach, and nasopharyngeal cancers [31], has reduced in Taiwan. This decline is attributable to the successful nationwide screening programs launched by the Taiwan government for cervical, oropharyngeal, colon and rectal cancers, and nationwide HBV vaccination. By contrast, no universal screening program has been implemented in Taiwan for lung cancer. Currently, lung cancer screening is included only as a part of the regular chest X-ray survey for pulmonary tuberculosis [38]. Therefore, the Taiwan government should consider promoting early diagnosis of potential high-risk individuals, for instance, implementing low-dose CT for high-risk patients with systemic diseases.

The major strengths of this study are the large number of included population-based cases and controls, high validity of the cancer diagnoses, and focus on health care services utilization. Nonetheless, the study has some limitations. First, we could not confirm early cancer diagnoses in patients with comorbidity based on their cancer stages because the NHIRD does not contain comprehensive data on cancer staging and treatment modalities in cancer patients; these data are recorded in the Taiwan Cancer Registry Database. Second, the period of 6 months before cancer diagnosis allowed for comorbidity diagnosis is insufficient for the monitoring of some chronic diseases; this may have limited the early cancer screening opportunities in some patients. Third, because of the inherent limitations of the NHIRD, information regarding the status of comorbid conditions was lacking.

In conclusion, different comorbid conditions will have unique effects and a given comorbidity can affect cancer care at multiple points in the cancer care decision making. We reported the significant outcome of lower mortality in lung cancer patients with comorbidity compared with those without comorbidity in 10 years after establishment of diagnosis. Patients with comorbidity required more frequent physician visits and greater opportunity to undergo screening which may assist in early cancer diagnosis. According to our findings, physicians should use caution regarding comorbidity in lung cancer patients, including regular monitoring of those with chronic conditions to facilitate early detection of lung cancer. The adverse consequences of comorbidity pose a major clinical challenge in the care of older cancer patients. Health services research that focuses on specific comorbidities and their effects in a cancer patient's clinical treatment decisions can produce new insights into the optimal diagnosis, treatment, and long-term surveillance of cancer patients with comorbid disease.

## Figures and Tables

**Table 1:** ICD-9-CM codes used to identify comorbidity.

Pulmonary diseases	Diagnosis	ICD-9-CM
No	Cerebrovascular accident	430, 431, 432.X, 433.X, 434.X, 434.X, 435.X, 436, 437.X, 438.X
	Coronary artery disease	410.X, 411.X, 412, 413.X, 414.X
	Diabetes mellitus	250, 357.2, 362.X, 366.41
	Disorders of adrenal glands	255.X
	Disorders of thyroid gland	240.X-246.X
	Duodenal ulcer	532.X
	Gastric ulcer	531.X
	Gastrojejunal ulcer	534.X
	Hyperlipidemia	272.X
	Hypertension	362.11, 401.X–405.X, 437.2
	Hypoparathyroidism	252.X
	Peptic ulcer	533.X

Yes	Asthma	493.X
	Bronchiectasis	494.X, 496
	Chronic bronchitis	491.X
	Emphysema	492.X
	Empyema	510.X
	Pulmonary tuberculosis	011.X

**Table 2:** Characteristics of lung cancer death subjects between 1995 and 2010 (*n* =86,990).

**Comorbidity**	**With comorbidity**		**Without comorbidity**		***p*-value**
**Characteristics**	**(N=38,608)**		**(N=48,382)**		
	No.	%^a^	No.	%^a^	
Gender					< 0.0001
Female	12,019	31.13%	13,924	28.78%	
Male	26,589	68.87%	34,458	71.22%	
Period of diagnosis (years)					0. 0018
1995-2000	7,204	18.66%	9,383	19.40%	
2001-2005	13,752	35.62%	17,412	35.99%	
2006-2010	17,652	45.72%	21,587	44.62%	
Diagnostic age (yrs)					< 0.0001
<=39	436	1.13%	1,552	3.21%	
40-49	1,537	3.98%	4,842	10.01%	
50-59	4,387	11.36%	9,225	19.07%	
60-69	8,835	22.88%	11,969	24.74%	
>=70	23,413	60.64%	20,794	42.98%	
Urbanization level					< 0.0001
1	12,742	33.00%	17,476	36.12%	
2	20,216	52.36%	25,304	52.30%	
3	3,607	9.34%	3,701	7.65%	
4	1,491	3.86%	1,456	3.01%	
5	552	1.43%	445	0.92%	
Geographic region					< 0.0001
Central	9,784	25.34%	10,581	21.87%	
Northern	17,398	45.06%	23,064	47.67%	
Eastern	903	2.34%	842	1.74%	
Southern	10,483	27.15%	13,837	28.60%	
Islands	40	0.10%	58	0.12%	

^a^ May not total 100% due to rounding. ^b^(*e*^ln⁡(*RR*)−1.96*SE*[ln⁡(*RR*)]^, *e*^ln⁡(*RR*)+1.96*SE*[ln⁡(*RR*)]^).

## Data Availability

The data used to support the findings of this study are available from the corresponding author upon request.
